# Ultrasonic Manipulation of Hydrodynamically Driven Microparticles in Vessel Bifurcation: Simulation, Optimization, Experimental Validation, and Potential for Targeted Drug Delivery

**DOI:** 10.3390/mi15010013

**Published:** 2023-12-21

**Authors:** Saqib Sharif, Daewon Jung, Hiep Xuan Cao, Jong-Oh Park, Byungjeon Kang, Eunpyo Choi

**Affiliations:** 1 School of Mechanical Engineering, Chonnam National University, Gwangju 61186, Republic of Korea; saqibaan@gmail.com; 2 Korea Institute of Medical Microrobotics, Gwangju 61011, Republic of Korea; jungdaewon@kimiro.re.kr (D.J.); hiep.caoxuan@kimiro.re.kr (H.X.C.); jop@kimiro.re.kr (J.-O.P.); 3 College of AI Convergence, Chonnam National University, Gwangju 61186, Republic of Korea; 4 Graduate School of Data Science, Chonnam National University, Gwangju 61186, Republic of Korea

**Keywords:** ultrasonic manipulation, acoustic radiation force, targeted drug delivery

## Abstract

Microrobots driven by multiple external power sources have emerged as promising tools for targeted drug and stem cell delivery in tissue regeneration. However, navigating and imaging these devices within a complex colloidal vascular system at a clinical scale is challenging. Ultrasonic actuators have gained interest in the field of non-contact manipulation of micromachines due to their label-free biocompatible nature and safe operation history. This research presents experimentally validated simulation results of ultrasonic actuation using a novel ultrasonic transducer array with a hemispherical arrangement that generates active traveling waves with phase modulation. Blood flow is used as a carrier force while the direction and path are controlled by blocking undesirable paths using a highly focused acoustic field. In the experiments, the microrobot cluster was able to follow a predefined trajectory and reach the target. The microrobot size, maximum radiation pressure, and focus position were optimized for certain blood flow conditions. The outcomes suggest that this acoustic manipulation module has potential applications in targeted tumor therapy.

## 1. Introduction

Although there have been remarkable advancements in cancer treatments, cancer remains the second leading cause of death [1]. Traditional therapies, such as chemotherapy, radiation therapy, and photodynamic therapy (PDT), have limitations. Chemotherapy exposes the entire body to high concentrations of toxic drugs, which can damage healthy tissue in the process of killing cancer cells. Moreover, radiation therapy can cause severe side effects due to radiation exposure, and PDT is expensive and challenging in terms of targeting cancer tissue for photosensitizing [2,3]. Despite its drawbacks, chemotherapy remains the most frequently used method for cancer treatment, with various classifications of anti-cancer drugs available. One of these is doxorubicin (DOX), which is a widely used, broad-spectrum anti-cancer agent with a strong effect on solid tumors. DOX is a hydrophilic anthracycline antibiotic that destroys the DNA double helix when inserted into the DNA of cancer cells. However, it also has negative effects on normal cells and can cause cardiac toxicity depending on the cumulative dose [4]. Therefore, it is crucial to minimize the toxicity of DOX to normal tissues without compromising its ability to kill cancer cells.

Targeted drug delivery (TDD) can precisely deliver anti-cancer drugs to infected tissues, and has emerged as a prominent solution to the toxicity problem [5,6]. A wide variety of nanoscale and microscale particles have been utilized as TDD carriers. Moreover, recent advancements in nanotechnology and three-dimensional (3D) printing have facilitated the on-demand fabrication of these microscale/nanoscale carriers, including both organic and inorganic materials. Some are created from metals, and others from polymers [7,8,9,10,11]. This technology offers promising potential for completing tasks in a variety of media and can be remotely controlled through the use of external power sources, including magnetic, electric, hydrodynamic, acoustic, and optical fields [12,13,14,15,16]. Each of these approaches has its own unique benefits and drawbacks. For example, optical manipulation provides precise control with high spatial resolution, although it has limited penetration depth and could cause harm to living objects [17,18,19,20]. Although magnetic manipulation offers powerful torque and greater penetration depth, it requires a specific setup and lacks selective control [21,22,23,24,25]. The electric method allows for a wide range of manipulable particle sizes, although it requires specific environmental conditions and particle materials. Moreover, the electrical power delivered into biological systems is limited to a certain skin depth and has severe side effects in high-conductivity environments [26,27,28]. The hydrodynamic manipulation technique is probably the simplest method of manipulating microparticles and nanoparticles by using fluid flow control within a microchannel. However, this approach requires a specific microchannel platform with a flow control method for a specific object, and the controllability of particle navigation is very limited [29,30,31]. A detailed comparison of these navigation schemes is given in Table 1.

Of the several methods available for the acoustic navigation of microparticles, acoustic manipulation has emerged as the prominent choice, particularly for applications in the domain of medical micromachines. This approach has notable advantages that originate from its unique characteristics. First, acoustic manipulation leverages the inherent ability of acoustic energy to traverse the human body with remarkable depth, allowing it to reach all organs and tissues. This feature endows it with unparalleled access to DDS applications. Second, the versatility of acoustic manipulation is underscored by its capacity to trap objects spanning the nanometer to millimeter scale at precise locations. This level of precision allows for the aggregation or separation of objects without necessitating specific material properties or characteristics, enhancing applicability in diverse scenarios. Moreover, the historical use of acoustic energy spans a considerable period, highlighting its well-established safety profile in terms of human interactions. Notably, it serves as a biocompatible energy source, aligning seamlessly with biomedical and clinical requirements with minimal risk to the human body. Accordingly, the use of acoustic manipulation in the context of microparticle navigation offers a compelling avenue for biomedical applications, leveraging its ability to penetrate the body, its versatility in object manipulation, and its established safety and biocompatibility credentials [32,33,34,35].

In terms of practical implementation, acoustic actuation typically employs one or more piezoelectric transducers. These transducers generate a pressure field that effectively entraps and manipulates the target object within low acoustic impedance media. This technique is commonly referred to as “acoustic tweezers,” which serve as a powerful tool for precise and controlled microparticle manipulation within certain fluidic environments [36,37,38,39]. However, when exposed to harsh hydrodynamic conditions, these traps are sufficiently strong to hold against dynamic fluidic flows in complex vascular systems. In addition, the number of particles that are trapped is also limited, rendering it a very passive method for delivering high doses of drugs.

**Figure 1:**
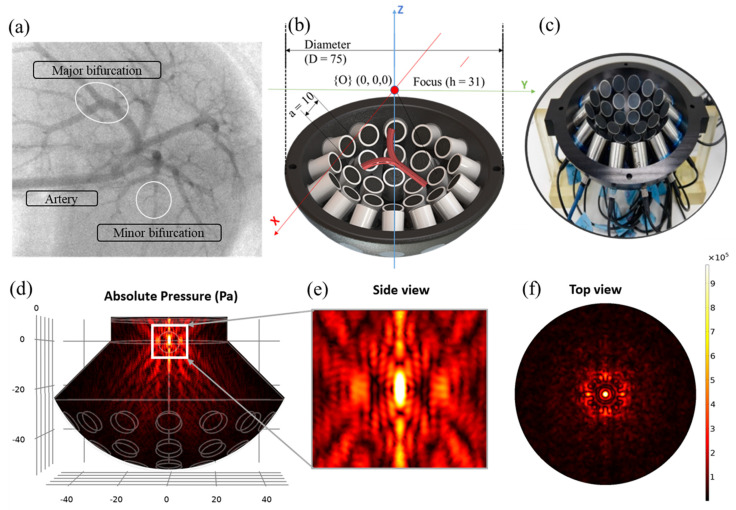
(**a**) Complexity of endovascular navigation in the portal vein of a rat. (**b**) Animated description of Y-shaped blood vessel bifurcation surrounded on the top of a 30 UT array ultrasonic actuator. (**c**) Photographic image of US actuator. (**d**) Absolute acoustic radiation pressure field. (**e**) Absolute acoustic radiation pressure field in the ZY zoomed plane. (**f**) Top view, absolute acoustic radiation pressure field in the XY plane.

In this article, we present a hybrid hydrodynamic manipulation system with external acoustic actuation. The primary carrier force is fluidic hydrodynamic drag, and the acoustic pressure force is then used to direct the microrobot along a designated path. Our simulation employed an acoustic actuator comprising 30 ultrasonic transducers operating at 1 MHz, and was able to produce both a single focused point and a twin trap for particle manipulation. Initially, the acoustic focus position was optimized to achieve efficient navigation, and the trajectories of the microrobots were then compared and optimized for efficient navigation under various vessel and fluid conditions and for different blood flow rates and carrier sizes. A detailed animated description of Y-shaped blood vessel bifurcation on the top of a 30 UT array ultrasonic actuator and the acoustic pressure generated at the focus position is given Figure 1.

## 2. Analytical Model

Considering a microsphere with a radius *r* submerged within a fluid flowing through a Y-shaped cylindrical channel, various forces would act upon this microparticle. These forces encompass the acoustic radiation force ($F_{Rad}$), the drag force exerted by the blood’s hydrodynamics ($F_{d}$), and the contact force arising from interactions between microparticles and the vessel wall ($F_{c}$). The translational motion of the particle can be mathematically described as follows:

(1) $$m_{p}\frac{d\vec{v}}{dt}=\vec{F_{Rad}}+\vec{F_{d}}+\vec{F_{c}},$$

where v is the translational velocity and $m_{p}$ is the particle mass.

### 2.1. Hydrodynamic Drag Force

We adopted a low-velocity approximation for a non-turbulent creeping flow scenario, assuming the incompressibility of blood. Employing the Stokes flow model, we expressed the hydrodynamic drag force ($F_{d}$) acting upon a spherical entity with a radius “r” submerged in the fluid as follows:

(2) $$\vec{F_{d}}=(\frac{1}{\tau_{p}})m_{p}\left(\vec{u}-\vec{v}\right),$$

(3) $$\tau_{p}=\frac{\rho_{p}\;d_{p}^{2}}{18\eta }.$$

where $m_{p}$ is the particle mass; $\tau_{p}$ is the particle velocity response time; u and v are the velocities of the fluid and particle, respectively; η is the dynamic viscosity of the fluid; and $d_{p}$ is the diameter of the microparticles. For a flow velocity ranging from 1 mm/s to 4 mm/s, the corresponding Reynolds number (Re) was calculated to range from 2 to 9, respectively, satisfying the laminar flow condition (Re < 10).

### 2.2. Force Acoustic Force

To compute the acoustic field, we used a far-field piston source with single-frequency emission controlled through acceleration. The complex acoustic pressure (*P*) generated by the entire array at a specific location resulted from the vector summation of 30 individual complex acoustic pressures (*P_j_*), with each piston source emitting at a single frequency. This phenomenon can be mathematically expressed as follows [40]:

(4) $$P=\sum_{j=1}^{30}P_{j}(r)=P_{0}A\frac{D_{f}(\theta )}{d}e^{i(\varphi +kd)},$$

(5) $$D_{f}=\frac{2J_{1}(aksin\theta )}{aksin\theta }.$$

where $P_{0}$ is the transducer amplitude constant power, *A* is the peak-to-peak amplitude of the excitation signal, $D_{f}$ is a far-field directivity function that depends on the angle *θ* between the transducer normal and point *r*, and *d* is the propagation distance in free space. Term ϕ is the phase delay of the transducer, k=2π/λ is the wavenumber, $J_{1}$ is a first-order Bessel function, and *a* is the radius of the emitting source.

When particles pass through this acoustic pressure field, they observe a dominant deflective force in that region, which is the acoustic radiation force (ARF). This ARF $({\overset{⃑}{F}}_{rad}),$ acting upon a diminutive spherical particle, can be calculated by leveraging the gradient of the Gor’kov potential field (*U*) [41,42].

(6) $${\overset{⃑}{F}}_{rad}=-\nabla U,$$

(7) $$\nabla^{2}U=\frac{\partial^{2}U}{\partial^{2}x}+\frac{\partial^{2}U}{\partial^{2}y}+\frac{\partial^{2}U}{\partial^{2}z},$$

(8) $$U=2K_{1}({\left|p\right|}^{2})-2K_{2}({\left|p_{x}\right|}^{2}+{\left|p_{y}\right|}^{2}+{\left|p_{z}\right|}^{2}),$$

(9) $$K_{1}=\frac{1}{4}V(\frac{1}{c_{0}^{2}\rho_{0}}-\frac{1}{c_{1}^{2}\rho_{1}}),$$

(10) $$K_{2}=\frac{3}{4}V(\frac{\rho_{0}-\rho_{1}}{\omega^{2}\rho_{0}(\rho_{0}+2\rho_{1})}),$$

where *V* is the volume of the microparticle, *ω* is the angular frequency of the emitting source acoustic wave, ρ is the density, and *c* is the speed of sound (with subscripts 0 and 1 referring to the host medium and the particle material, respectively). Term *p* is the complex pressure, while $p_{x},\;p_{y},\;and\;p_{z}$ are its derivatives over the X-, Y-, and Z-directions, respectively. The density and speed of sound of polystyrene microparticles are 1050 kg/m^3^ and 1500–1600 m/s, respectively [43,44,45], whereas the density and speed of sound of water are 997 kg/m^3^ and 1450 m/s, respectively.

## 3. Results and Discussion

For the investigation, we employed COMSOL Multiphysics^®^ software (version 5.4, COMSOL Inc., Burlington, MA, USA) to simulate and analyze the following relevant acoustic phenomena: acoustic fields, acoustic radiation force, and interactions between particles and a fluid medium within a vessel bifurcation channel. Initially, a computational simulation was conducted to model an array of 30 ultrasonic transducers (UTs) operating at a resonance frequency of 1 MHz, along with a 3D manipulation control technique [46]. Subsequently, the resultant acoustic fields were integrated into our vessel’s geometric configuration using the particle tracing for fluid flow (fpt) module within the COMSOL software. The details of the simulation’s parameters are provided in Table 2.

**Figure 2:**
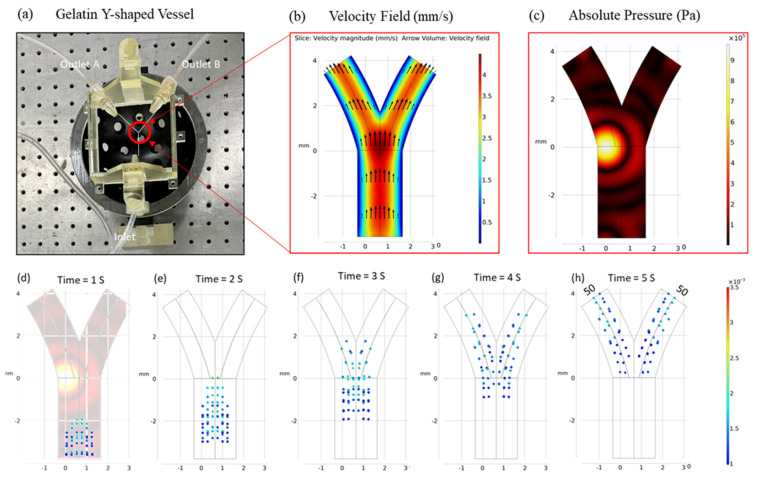
(**a**) Experimental model of 3D-printed gelatin Y-shaped micro-vessel. (**b**) Two-dimensional cross-sectional projection of fluid velocity in the vessel. (**c**) Cross-sectional representation of focused acoustic pressure in bifurcation vessel. (**d**–**h**) Particle trajectories in the absence of any external acoustic field exhibited a state of uniform particle distribution within both channels. The fluid’s mean velocity and particle diameter were established as 1 mm/s and 10 µm, respectively.

The vessel geometry under consideration was a simple Y-shaped bifurcation that featured inlet and outlet diameters of 3.0 and 1.5 mm, respectively. It should be noted that the average fluid velocity (*v*) at the inlet was a controllable parameter, allowing for adjustments that would subsequently influence the velocity distribution within the vessel. A non-pulsatile, steady, creeping flow of a weakly compressible fluid was also assumed. The velocity profile across the vessel’s cross-section is visually presented in Figure 2b.

In this simulation, an ensemble of 100 micro-particles was introduced at the inlet, each possessing an initial velocity of “v.” These particles were released sequentially in four temporal steps, with 25 particles released in each step. The particle diameters ranged from 5 to 20 µm, and their acoustic properties (such as compressibility and speed of sound within the medium) remained consistent across all simulations. Figure 2d–h illustrate the behavior of these particles in the absence of an external acoustic pressure field, where they predominantly followed the fluid drag flow, resulting in a uniform dispersion within the vessel. Notably, 50 particles reached each outlet in a similar sequential pattern. The tracking of particle distribution at both outlets was facilitated using the particle counting feature within the COMSOL software.

After introducing the acoustic field, the particles exhibited altered trajectories in response to the gradient field. However, it is important to highlight that these trajectories were notably influenced by various factors, including the strength of the applied acoustic field, the position of focus within the vessel, the initial velocity of the particles and the carrier fluid, and the diameter of the individual particles.

Under specific conditions, the particles exhibited reduced velocity as they traversed the inner surface of the blood vessel. Although the study primarily considered minimal particle–wall interactions (limited to general reflections), the cumulative presence of static particles adhering to the vessel’s inner wall could not be disregarded. Consequently, to ensure effective particle navigation within the blood vessel while mitigating the risk of vessel blockage, the selection of an optimized focal position and an appropriate acoustic field tailored to the prevailing fluid flow conditions became imperative.

### 3.1. Optimized Focus Position

Optimizing the focus position of microparticles in ultrasound navigation is of paramount importance in the field of microscale robotics and biomedical applications. This optimization process involves precisely controlling the focus of ultrasound waves to manipulate and guide particles within a fluidic vessel medium. These microscale particles often have specific tasks, such as drug delivery, tissue sampling, or cellular manipulation. Moreover, optimizing the focus position allows for the precise control of the microparticles’ movements within the vessel’s geometry, ensuring that they reach their intended targets accurately. This is particularly critical for minimally invasive medical procedures. Precise focus optimization also reduces the energy requirements for manipulating microrobots and particles. This consideration assumes significance in terms of prolonging operational lifespans and mitigating the exposure of the human body to excessive external radiation. Notably, one of the most critical aspects of optimizing focus positions is preventing particles from adhering to the vessel wall, which could result in vessel blockage.

This part of the study involved conducting an investigation to assess the effects of varying focal spot positions on particle trajectories under different conditions. These conditions were manipulated by altering parameters such as fluid velocity, particle diameter, and acoustic pressure. The adjustments to the focal spot position were executed manually, and meticulous monitoring of particle trajectories was conducted to evaluate their efficacy in navigation and to ascertain whether the particles adhered to the vessel wall.

**Figure 3:**
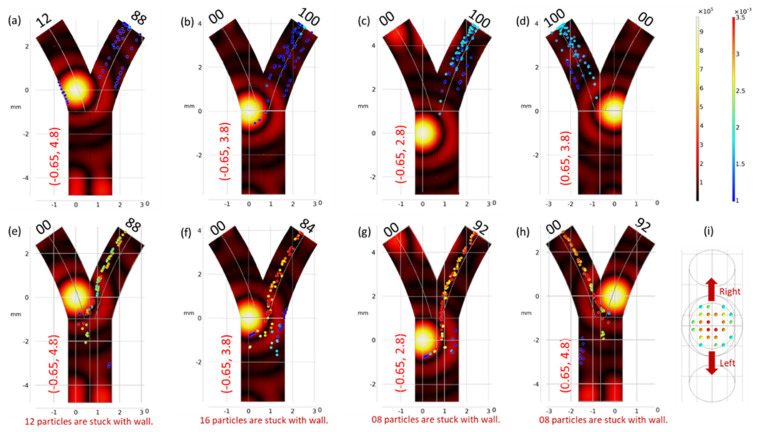
Trajectories of particles under the influence of the acoustic force gradient (∇p). The particles in this analysis had a fixed diameter (*d*) of 10 µm. The fluid velocity ($v_{avg}$) and the maximum acoustic pressure (${acpr}_{max\;}$) were set at 1 mm/s and 2 MPa for cases (**a**–**d**), and at 2 mm/s and 2 MPa for cases (**e**–**h**), respectively. In cases (**a**–**c**) and (**e**–**g**), the ∇p force directed the particles towards the right bifurcation. Conversely, in cases (**d**) and (**h**), the particles exhibited movement in the opposite direction (i.e., towards the left bifurcation). (**i**) Initial release position of the particles.

The findings of the investigation revealed that the optimal focal spot position is contingent upon the prevailing external conditions. This observation underscores the critical importance of accounting for this phenomenon when developing a model for an effective navigation system. Detailed results and insights into this phenomenon are presented in Figure 3. It should be noted that in Figure 3h, which replicates Figure 3e in the opposite direction, the particles moving in the left direction are slightly different from the particles moving in the right direction. Further investigations revealed that this was due to the non-uniform initial release position of the particles, as depicted in Figure 3i.

### 3.2. Velocity Correction

**Figure 4:**
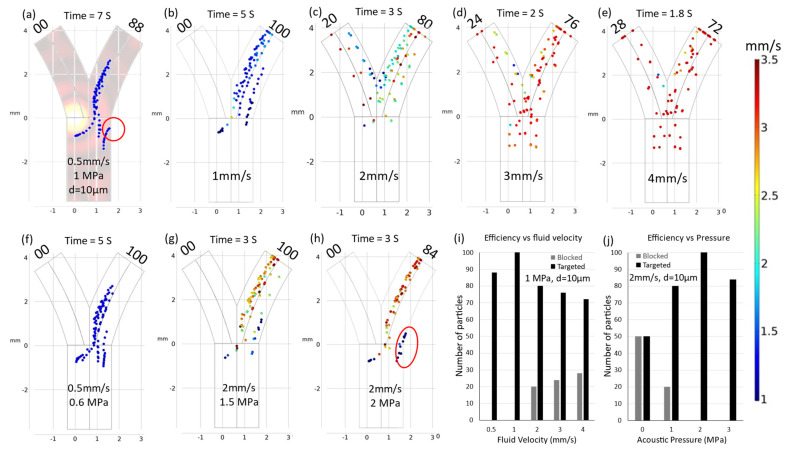
Trajectories of particles. (**a**) Particles adhered to vessel wall as shown in the red circle when the fluid velocity was decreased. (**b**–**e**) Micro-particle navigation efficiency depended on increasing fluid velocity. (**f**) Particle adhesion to the vessel wall was reduced by decreasing acoustic pressure. (**g**) The efficiency was increased to 100% by increasing acoustic pressure to 1.5 MPa. (**h**) Navigation efficiency decreased with increasing pressure due to particles sticking to the vessel wall as shown in red circle. (**i**) Efficiency’s dependence on fluid velocity. (**j**) Efficiency’s dependence on acoustic pressure.

Fluid flow represents a challenging parameter in the context of intravascular navigation systems due to its inherent complexities. This challenge primarily arises from the predominantly non-uniform nature of flow patterns within in vivo systems, rendering precise control difficult. Variations in fluid velocity within vascular conduits cause alterations in drag forces, exerting a profound influence on the navigation dynamics of microparticles. A graphical representation of this phenomenon is provided in Figure 4.

Under specific conditions, notably at an acoustic pressure (*acpr*) of 1MPa and a particle diameter (*d*) of 10 µm, optimal navigation was achieved at a fluid velocity (*v*) of 1 mm/s. However, deviations from this velocity (increases or decreases) could result in a suboptimal performance of the navigation system.

When the average fluid velocity was augmented, as depicted in Figure 4b–e, the particles experienced an acceleration in the presence of escalating drag forces and spent less time under the influence of acoustic radiation. This situation increased the probability of particles penetrating through the vessel and caused a linear reduction in navigation efficiency, which was proportional to the increases in velocity.

Conversely, when the average fluid velocity diminished, the particles exhibited reduced mobility within the carrier fluid, prolonging their exposure to acoustic radiation pressure. This phenomenon forced the particles toward the vessel wall, culminating in particle adhesion and potential blockage. Therefore, it is imperative to recognize that fluid velocity assumes a pivotal role in the modeling of microrobot and particle navigation. Moreover, precise modulation of fluid velocity holds the key to achieving targeted delivery and navigation, establishing it as an indispensable consideration in the development of effective microscale navigation systems.

Strategic optimization of acoustic pressure values is one method of addressing the challenge of varying fluid velocity. To illustrate this approach, consider an initial scenario in which the fluid velocity (*v*) has been reduced to 0.5 mm/s, resulting in a 24% decrease in navigation efficiency and the adherence of 12 particles to the vessel wall. This issue is mitigated by adjusting the acoustic pressure values. By decreasing the acoustic pressure to 0.5 MPa, a subset of particles becomes able to discover a passage through the vessel. In contrast, increasing the acoustic pressure to 0.6 MPa results in achieving a 100% navigation efficiency rate.

**Figure 5:**
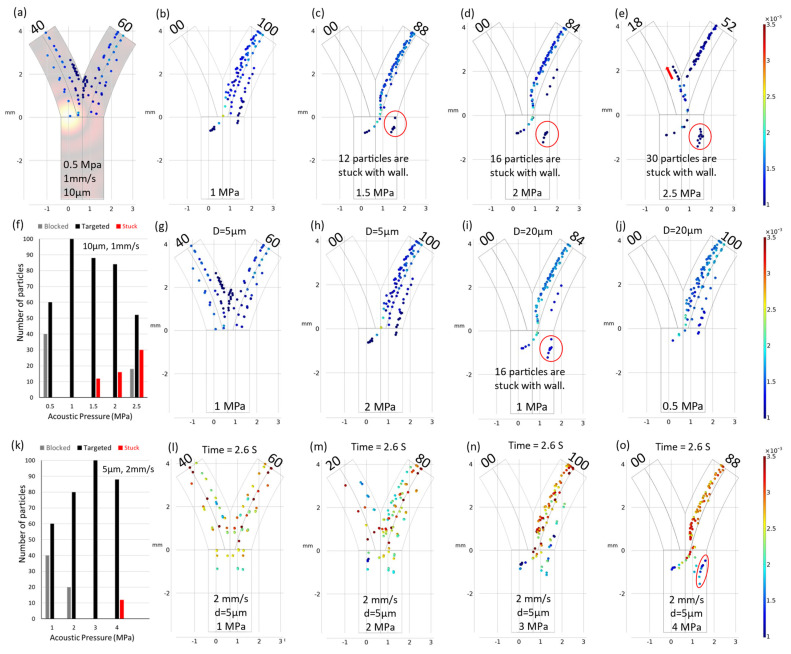
(**a**–**e**): Trajectories of particles, illustrating the change in navigation and particles in red circles demonstrate the adhesion with increasing acoustic pressure. Particle adhesion to the vessel wall is a consequence of elevated acoustic pressure beyond 1 Mpa. When increased further, secondary deviation of particles in the opposite direction was observed as described by the arrow. (**f**) Efficiency dependence on acoustic pressure for 10 µm particles in a low-velocity fluid environment (velocity = 1 mm/s). (**g**) Decrease in navigation efficiency correlated with diminishing particle size. (**h**) Restoration of 100% navigation efficiency through an increase in acoustic pressure. (**i**) Illustration of particle adhesion to the vessel wall resulting from an increase in particle size. (**j**) Enhancement in efficiency achieved with a reduction in acoustic pressure to 0.5 MPa. These trajectories exemplify the critical influence of particle size and acoustic pressure on microparticle navigation dynamics. (**k**) Efficiency dependence on acoustic pressure for 5 µm particles in a high-velocity fluid environment (velocity = 2 mm/s). (**l**–**o**) Trajectories of particles with decreased particle sizes (d = 5 µm) and increased velocity (2 mm/s).

In the second scenario, when the particle velocity was increased to 2 mm/s, there was a consequent 40% reduction in efficiency, as depicted in Figure 4c. To rectify this situation, the acoustic pressure value was incrementally increased to 2 MPa, effectively blocking all particle movement in the left direction while simultaneously forcing particles toward the vessel wall. This action resulted in 16 particles adhering to the vessel wall, as demonstrated in Figure 4h. Subsequently reducing the pressure to 1.5 MPa facilitated the attainment of 100% navigation efficiency, as depicted in Figure 4g. These simulated trajectories highlight the critical importance of optimal acoustic pressure values in accordance with specific fluidic and particle conditions to ensure effective navigation. Figure 5 provides additional insights into the effect of minor adjustments to acoustic pressure on both navigation efficiency and the occurrence of particle adhesion to the vessel wall. Notably, an increase in acoustic pressure to 2.5 MPa induced a deviation in particle trajectories within the fringing field. This phenomenon is elucidated in Figure 5e, where the influence of a secondary fringing field is exemplified, which resulted in the redirection of 18 particles in the opposite direction.

### 3.3. Particle Size Dependence

When modeling any navigation system for microparticles, the importance of particle size cannot be ignored, because the efficacy of ultrasonic navigation for microparticles is precisely dependent on particle size. The sizes of microparticles significantly influence their response to both acoustic and hydrodynamic drag forces, determining their mobility and maneuverability within fluidic environments. Smaller particles exhibit heightened sensitivity to manipulation, allowing for precise control and targeting. This is particularly important in drug delivery applications, where navigating through intricate microanatomy is paramount. Conversely, larger particles experience increased drag forces, as well as acoustic force, and could be more prone to adhesion, potentially limiting their navigational capabilities and presenting vessel blockage concerns. Therefore, understanding and optimizing particle size is a fundamental consideration in the development of effective ultrasonic navigation strategies and can affect the success and versatility of microscale applications.

In the previously discussed navigation scenarios, the particle diameter (*d*) remained constant at 10 µm. However, when the particle diameter was reduced to 5 µm, as depicted in Figure 5g, a substantial reduction of 80% in navigation efficiency was observed. This significant efficiency decrease was primarily due to the diminished acoustic force exerted on smaller particles. It should be noted that drag and acoustic forces are size-dependent, with the drag force linearly proportional to particle diameter ($F_{d}\;\propto d$) and the acoustic force scaling with the cube of the diameter ($F_{acpr}\propto d^{3}$). The decline in efficiency due to reduced particle size was ameliorated by increasing the acoustic pressure. When the acoustic pressure was increased to 2 MPa under the same conditions, a return to 100% efficiency was achieved. Conversely, changing the particle size without any corresponding adjustments of acoustic pressure resulted in particle–wall adhesion, which was effectively mitigated by reducing the acoustic pressure, as demonstrated in Figure 5i.

### 3.4. Experimental Validation

**Figure 6:**
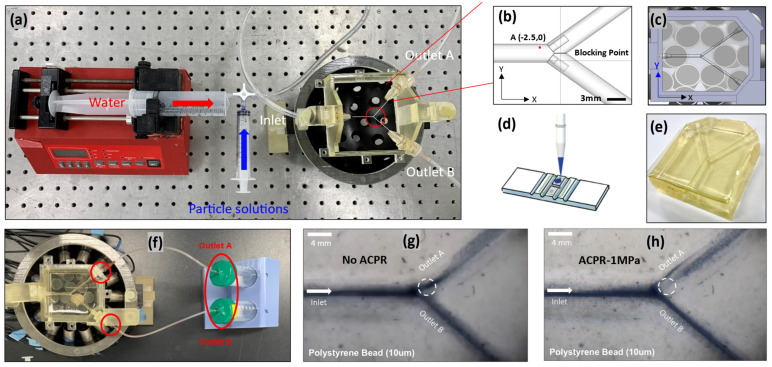
(**a**) Top experimental view of the fluid channel with specified inlet and outlets. (**b**,**c**) Animated description of the channel. (**d**) Hemocytometer counting chamber. (**e**) Gelatine-based bifurcation channel used in the experiment. (**f**) Fluid channel suspended on top of the ultrasonic transducers. (**g**,**h**) Images of particles flowing inside the fluid channel with and without external acoustic field, respectively.

To validate the proposed simulations experimentally, an acoustic blocking experiment was conducted to verify the feasibility of the simulation method. For this blocking test, the following experimental setup was constructed. A schematic of the experiment is shown in Figure 6. The designed actuator was built with 30 immersion types, and each signal from the transducers was modulated by the custom control system. The particle flow line comprised a syringe pump and a particle receiver. DI-water and a 10 μm-polystyrene microbead solution were inserted into the gelatin channel, where a flow rate of 2 mL/min was supplied by the pump. The targeting performance of the system was checked using the hemocytometer method. For the microbead solution used in the test, the targeting efficiency was 78.61% ± 0.21%.

Initially, the microchannel was tested for flow imperfections without applying any acoustic field. The results were satisfactory (48–52) and are displayed in Table 3. When an acoustic field was applied, the particles deviated towards channel-B. An efficiency of 21–79 particles was recorded, which was similar to the simulated results (20–80) displayed in Figure 4h.

## 4. Conclusions

The findings of this study underscore the significance of employing ultrasonic manipulation techniques within a bifurcated fluid channel. By directing focused acoustic pressure radiation to specific optimized positions, particles can be steered along the desired trajectories. It is evident from our observations that particle deviation is profoundly influenced by the selected focus position, highlighting the critical need to ascertain the optimal focal point prior to experimental implementation. Furthermore, our results demonstrated the necessity of tuning certain parameters (including the particle size and acoustic pressure) to match the prevailing flow conditions when conducting experiments. These observations were experimentally validated under conditions and parameters similar to those employed in the simulations. We also determined that the efficiency of particle navigation is intricately linked to variables such as carrier fluid velocity, fluid viscosity, and particle size. A discernible reduction in efficiency was noted with increasing fluid velocity, which could be ameliorated by adjusting the acoustic pressure settings. Collectively, these insights contribute to a more comprehensive understanding of the factors that govern particle manipulation within complex fluidic environments.

## Figures and Tables

**Table 1:** Overview of externally powered platforms utilized in the wireless manipulation of particles.

Actuation Power Platform	Particle Size (μm)	Input Power	Control Force ^1^	Limitations
Optical field [17,18,19,20]	0.1–100	10^6^–10^7^ (W/cm^2^)	Trapping force and torque (pN)	Limited skin depth, high-powered laser system.
Magnetic field [21,22,23,24,25]	0.1–10	1–10 (Tesla)	Magnetic gradient field force (nN to μN)	Particle size limitations, only works with magnetized particles.
Electric field [26,27,28]	0.001–1000	10^4^–10^7^ (V/m)	Dielectrophoresis force (pN to μN)	Low-conductivity media, ionization.
Hydrodynamic field [29,30,31]	0.1–1100	N/A	Hydrodynamic effects (pN to μN)	Flow control limitation, specific platform.
Acoustic field [32,33,34,35,36,37,38,39]	0.1–1000	10^−2^–10 (W/cm^2^)	Axial acoustic force (μN)	Low acoustic impedance media, trap is too weak in strong flows.

^1^ The range of control force is given for specialized conditions.

**Table 2:** Simulation parameters.

Name	Expression	Value
Fluid viscosity	$\eta_{f}$	$5\times 10^{-3}\;\left[Pa\cdot s\right]$
Fluid density	$\rho_{f}$	$997\;\left[kg\cdot m^{-3}\right]$
Particle density	$\rho_{P}$	$1032\;\left[kg\cdot m^{-3}\right]$
Particle diameter	$d_{p}$	$10\;\left[µm\right]$
Vessel diameter	D	$2\;\left[mm\right]$
Average fluid velocity	$v_{f}$	$1-4\;\left[mm\cdot s^{-1}\right]$
Number of UT array elements	*UT*	30
Resonance frequency	$w_{0}$	$1\;\left[MHz\right]$
Controllable voltage	$V_{PP}$	$10–200\;\left[V\right]$
UT array diameter	D	$75\;\left[mm\right]$
The distance from array surface to focal point	h	$31\;\left[mm\right]$

**Table 3:** Targeting efficiency in gelatin bifurcation channel with 10 µm polystyrene bead solution and 2 mL/min fluid flow.

No External Acoustic Pressure				BLOCKING-A			
Sample-1		Sample-2		Sample-1		Sample-2	
Channel_A	Channel_B	Channel_A	Channel_B	Channel_A	Channel_B	Channel_A	Channel_B
47	48	42	56	23	88	19	68
53	50	54	43	16	44	14	79
40	47	62	58	16	64	20	85
49	57	45	63	17	72	31	73
472,500	505,000	507,500	550,000	180,000	670,000	210,000	762,500
48.34	51.66	47.99	52.01	21.18	78.82	21.59	78.41
48.16	0.17	51.84	0.17	21.39	0.21	78.61	0.21
CH_A: 48.16% ± 0.17%		CH_B: 51.84% ± 0.17%		CH_A: 21.39% ± 0.21%		CH_B: 78.61% ± 0.21%	

## Data Availability

The data that support the findings of this study are available from the corresponding authors upon reasonable request.
